# Viral Burden and Illness Severity During Acute SARS-CoV-2 Infection Predict Persistent Long COVID Symptoms

**DOI:** 10.1093/ofid/ofaf048

**Published:** 2025-01-30

**Authors:** Elisabeth Brandstetter Figueroa, Anne E P Frosch, Kristina S Burrack, Gayathri Dileepan, Rachael Goldsmith, Morgan Harris, Nwando Ikeogu, Hodan Jibrell, Sangeitha Thayalan, Robin L Dewar, Chetan Shenoy, Irini Sereti, Jason V Baker

**Affiliations:** Division of Infectious Diseases, Hennepin Healthcare Research Institute, Minneapolis, Minnesota, USA; Division of Epidemiology and Community Health, School of Public Health, University of Minnesota, Minneapolis, Minnesota, USA; Division of Infectious Diseases, Hennepin Healthcare Research Institute, Minneapolis, Minnesota, USA; Department of Medicine, School of Medicine, University of Minnesota, Minneapolis, Minnesota, USA; Division of Infectious Diseases, Hennepin Healthcare Research Institute, Minneapolis, Minnesota, USA; Center for Immunology, University of Minnesota, Minneapolis, Minnesota, USA; Division of Infectious Diseases, Hennepin Healthcare Research Institute, Minneapolis, Minnesota, USA; Division of Infectious Diseases, Hennepin Healthcare Research Institute, Minneapolis, Minnesota, USA; Division of Infectious Diseases, Hennepin Healthcare Research Institute, Minneapolis, Minnesota, USA; Division of Infectious Diseases, Hennepin Healthcare Research Institute, Minneapolis, Minnesota, USA; Division of Infectious Diseases, Hennepin Healthcare Research Institute, Minneapolis, Minnesota, USA; Division of Infectious Diseases, Hennepin Healthcare Research Institute, Minneapolis, Minnesota, USA; Frederick National Laboratory, Leidos Biomedical Research, Frederick, Maryland, USA; Department of Medicine, School of Medicine, University of Minnesota, Minneapolis, Minnesota, USA; Division of Intramural Research, National Institute of Allergy and Infectious Diseases, Bethesda, Maryland, USA; Division of Infectious Diseases, Hennepin Healthcare Research Institute, Minneapolis, Minnesota, USA; Department of Medicine, School of Medicine, University of Minnesota, Minneapolis, Minnesota, USA

**Keywords:** SARS-CoV-2, COVID-19, long COVID, post-acute sequelae of SARS-CoV-2 infection (PASC), viral burden

## Abstract

**Background:**

Long COVID is a common complication of infection with severe acute respiratory syndrome coronavirus 2, but the prevalence and predictors of the condition remain poorly characterized.

**Methods:**

We prospectively studied adults (≥18 years) with acute coronavirus disease 2019 (COVID-19) presenting to an urban safety net hospital and associated clinics between July 2020 and December 2022. Logistic regression models were used to evaluate the association between baseline demographic, clinical, and laboratory characteristics with long COVID status, defined as symptoms persisting at least 9 months after acute disease. Among unrecovered participants, we describe the prevalence of individual symptoms.

**Results:**

We enrolled 222 participants, 162 (73%) of whom had known recovery status by 9 months. Median age was 54 years, half (55%) were female, and the majority of participants (78%) had at least 1 comorbidity at the time of COVID-19 diagnosis. Based on acute illness characteristics, the adjusted odds ratio for long COVID was 3.0 (95% confidence interval [CI], 1.1–8.0) among those with detectable nucleocapsid antigen and 3.6 (95% CI, 1.2–11) for those who required supplemental oxygen. Of the 41% of participants with symptoms persisting at least 9 months, central nervous system and psychological symptoms were most commonly reported, with 57% reporting functional limitations due to their persistent symptoms.

**Conclusions:**

The strong association with initial disease suggests a decreasing prevalence of long COVID as acute illnesses become milder. However, many contemporary patients still experience high viral burden with extended viral replication, even after vaccination. Our findings highlight the importance of properly characterizing long COVID as viral evolution shifts acute disease presentation.

Long COVID is a common complication of severe acute respiratory syndrome coronavirus 2 (SARS-CoV-2) infection, characterized by persistent symptoms involving multiple organ systems following initial recovery of the acute illness phase. More than 60 symptoms have been associated with long COVID, including systemic symptoms, upper respiratory, cardiopulmonary, gastrointestinal, and neuropsychological [1]. Additional terms used to describe the condition include post-COVID conditions, long-haul COVID, or postacute sequelae of SARS-CoV-2 infection.

Multiple case definitions for long COVID have been proposed and circulated since the onset of the pandemic from the United States (US) Centers for Disease Control and Prevention [2], the United Kingdom’s National Institute for Health and Care Excellence [3], the World Health Organization [4], and the US RECOVER study [5], with varying acknowledgment of individual symptoms, requirements for minimum duration, and disease severity. Only in August 2024 was a comprehensive definition of the disease proposed. The new National Academies of Sciences, Engineering, and Medicine (NASEM) definition requires a symptom duration of at least 3 months, but acknowledges that these symptoms may be delayed, relapsing, remitting, or progressive, and may arise from multiple organ systems [6, 7].

Until the new definition was recently adopted, the heterogeneity in terminology, case definitions, and clinical manifestations have hindered progress in accurately describing long COVID prevalence and developing prevention and treatment strategies. Moreover, the inconsistent definitions have complicated research on underlying mechanistic causes, though clinical characteristics such as prior vaccination, acute coronavirus disease 2019 (COVID-19) disease severity, comorbidities (eg, diabetes, hypertension), and the inflammatory response to acute disease have been implicated as associated with long COVID risk [8–11].

Among a cohort prospectively enrolled with acute COVID-19 illness in both the pre- and Omicron eras, we aim to describe the prevalence of long COVID defined by symptoms persisting at least 9 months (corresponding to the end of the 6-month and beginning of the annual consecutive visit windows in our study). We also describe the spectrum of symptoms and explore potential clinical, virologic, and immunologic predictors of long COVID.

## METHODS

### Study Design and Participants

This study is a prospective cohort of adults (≥18 years of age) with acute COVID-19 illness presenting for care at an urban safety net hospital and associated clinics (Hennepin Healthcare, Minneapolis, Minnesota) from July 2020 through December 2022.

Eligible participants had a positive rapid antigen or reverse-transcription polymerase chain reaction diagnostic test for SARS-CoV-2, were within 15 days of diagnosis, and spoke English or Spanish. Baseline assessments were conducted at enrollment: a blood specimen was collected, and demographic and clinical characteristics were abstracted from the electronic medical record and supplemented by participant history. Participants returned for follow-up visits at months 1, 3, 6, and 12, and annually thereafter. The analysis cohort consisted of participants enrolled prior to 31 December 2022, and all participants had passed their annual visit window (defined as 9–18 months) by the time of analysis.

Candidate participants who were hospitalized were identified through positive tests at admission throughout the entire study period, whereas outpatient candidates were recruited starting in October 2021 by sending study information to patients with positive tests via electronic medical records messages (EPIC, Verona, Wisconsin). All participants provided written informed consent to participate in the research, which was approved by the Hennepin Healthcare Research Institute Institutional Review Board (Study ID: 20-4824). Study data were managed using REDCap electronic data capture tools [12, 13].

### Symptom and Recovery Assessments

Participants’ recovery status and the presence of symptoms persisting after acute illness were ascertained using a structured assessment questionnaire during follow-up visits (questionnaire available in the Supplementary Materials). Participants were asked if they had recovered or returned to their usual state of health (prior to their COVID-19 illness) with the following response options provided: completely recovered (no lingering symptoms and can conduct usual activities without limitations), mostly, somewhat, or not at all recovered. The month corresponding to symptom resolution was ascertained from those who reported complete recovery. Participants who reported not being recovered and were still having active symptoms were asked about the presence/absence and severity of individual symptoms, categorized as upper respiratory, systemic, cardiopulmonary, central nervous system (CNS)/psychological, gastrointestinal, and dermatologic symptoms. Finally, participants were asked the degree to which their symptoms caused functional limitations: interference with physical activity, work or job functions, activities of daily living (ADLs), and social interactions.

The symptom assessment questionnaire was implemented in June 2021 in response to early reports of persistent symptoms in the literature. Participants who enrolled in 2020 and early 2021 had their initial symptom assessments performed during their 6-month or annual visit window rather than shortly after acute illness (Supplementary Figure 1). For analyses, any symptoms reported as COVID-related at the first assessment time point were assumed to be present since acute illness. Conversely, when participants reported the absence of a symptom or complete recovery, this designation was carried forward through the duration of follow-up regardless of symptoms reported later.

Our primary outcome of interest was recovery status at 9 months after acute COVID-19 diagnosis, corresponding to the beginning of the annual visit window. Participants who indicated complete recovery prior to the annual visit, who had a symptom assessment performed during the annual visit window, or who had a symptomatic assessment completed after the annual visit window were included in the analysis. If multiple symptom assessments were completed in a subsequent visit window, the first assessment after the cutoff was used. Participants who were not recovered at a given timepoint and lacked subsequent symptom assessments were classified as lost to follow-up in future recovery windows. In sensitivity analyses, we also defined recovery and the presence of persistent symptoms at 3 and 6 months.

### Clinical Definitions and Blood Measurements

Comorbidities considered in analyses included a diagnosis of an immunocompromising condition, diabetes mellitus, hypertension, coronary heart disease or heart failure, chronic obstructive pulmonary disease, stroke, chronic kidney disease (stage 3 or greater), or obesity (body mass index [BMI] ≥30 kg/m^2^). Immunocompromising conditions included documentation or self-report of an autoimmune disorder, solid organ transplant, invasive cancer treatment in prior 6 months, human immunodeficiency virus, or use of immunosuppressant medications at time of COVID-19 diagnosis (excluding treatments for COVID-19 illness). These comorbidities were assessed individually and pooled together as a composite of “any comorbidity.” SARS-CoV-2 variants were imputed by using the predominant (>50%) circulating strain in Minnesota at the time of diagnosis (wild-type: 6 July 2020 to 28 February 2021; Alpha: 1 March to 5 July 2021; Delta: 6 July to 31 December 2021; Omicron lineages: 1 January to 31 December 2022) [14]. We also considered therapies utilized during acute illness among both inpatients and outpatients (antiviral: remdesivir, nirmatrelvir/ritonavir; immunomodulatory: corticosteroids, tocilizumab, baricitinib). Vaccination status at time of diagnosis was defined as follows: not vaccinated, primary series incomplete (1 dose of a messenger RNA [mRNA] vaccine), primary series complete (1 dose of Johnson & Johnson or 2 doses of an mRNA vaccine) and boosted (at least 1 additional dose beyond the primary series). Ultimately, vaccination status was dichotomized into “not vaccinated or primary series incomplete” and “primary series complete and/or boosted”).

Plasma specimens were processed from blood samples during acute illness. Quantitative plasma SARS-CoV-2 nucleocapsid antigen (N Ag) levels were measured using a microbead-based immunoassay (Quanterix), where circulating antigen >3 pg/mL (the lower limit of quantification) was considered detectable [15]. Anti-N pan-immunoglobulin was measured with the Bio-Rad Platelia SARS-CoV-2 Total Antibody Test where a normalized signal-to-cutoff (S/Co) ratio ≥1.0 was considered detectable, according to the manufacturer's directions. Anti-spike pseudo-neutralizing antibody was measured using a Genscript CPass SARS-CoV-2 Surrogate Virus Neutralization Test, which quantifies the percentage of binding that is inhibited by the participant's antibodies, ranging from 0 to 100%, and binding inhibition ≥30% was considered detectable (Supplementary Figure 2*A–C*) [16].

### Data Analysis

Demographic (age, sex, race/ethnicity), baseline characteristics (BMI, comorbidities, smoking status), acute COVID-19 illness characteristics (hospitalization status, supplemental oxygen requirement, vaccination status, acute therapies), and baseline laboratory measures (detectable N Ag, anti-N immunoglobulin G [IgG], and anti-S IgG) were summarized as counts and percentages for categorical variables and median and range for continuous variables. Among those with a known recovery status at 9 months, we described characteristics of those who were recovered versus not recovered. The prevalence of each reported symptom was summarized among those who had persistent symptoms past time points of 3, 6, and 9 months, respectively.

To investigate associations between demographic, clinical, and laboratory characteristics with recovery status at 9 months, logistic regression models were used to estimate odds ratios (ORs) and 95% confidence intervals (CIs). Covariates were considered in adjusted models as follows: model 1 was unadjusted; model 2 adjusted for age, sex, race/ethnicity, and the composite variable of “any comorbidity” described above; model 3 adjusted for highest oxygen requirement during acute illness in addition to all covariates included in model 2; and model 4 adjusted for detectable N Ag level in addition to all covariates included in model 3. Since measures of illness severity were highly correlated and also related to vaccine status and variant, we created a model of recovery at 9 months including hospitalization, oxygen requirement, immunomodulatory therapy during acute illness, vaccination status, variant, and acute detectable N Ag level. This informed the choice of including N Ag in model 4 as the strongest independent predictor.

In a sensitivity analysis, models 1 and 4 were repeated to estimate ORs and 95% CIs for persistent symptoms (not recovered) using alternative cutoffs of 3 and 6 months. Statistical inference was based on a 2-sided *P* < .05, and all analyses were performed using R Statistical Software (v4.1.0; R Core Team).

## RESULTS

**Figure 1.**
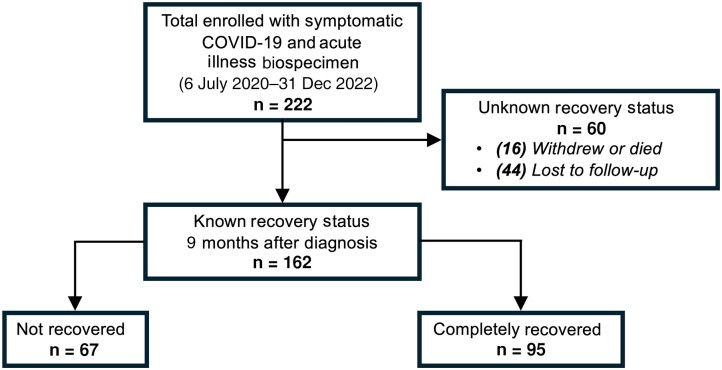
Study sample and recovery status for analysis cohort. The symptom assessment form was implemented on 21 June 2021 and, at that time, 100 of 222 were already enrolled into the cohort. Among the 95 completely recovered by 9 months, 72 had that designation made based on an assessment completed during their 1-, 3-, or 6-month visit, 20 from their annual visit (9–18 months), and 3 from a form completed after their annual visit. Among the 67 not recovered, 59 had that designation made based on an assessment completed during their annual visit and the remaining 8 from an assessment completed after their annual visit. Abbreviation: COVID-19, coronavirus disease 2019.

A total of 222 participants were enrolled with symptomatic acute COVID-19 illness between 6 July 2020 and 31 December 2022. Of those, 162 (73%) had a known recovery status by 9 months, corresponding to the start of the annual visit window (Figure 1). In comparison to those who did not have a symptom assessment after acute illness, the analysis cohort had a greater proportion of female, non-Hispanic White participants who were less likely to be hospitalized or have a pre-Omicron variant but were more likely to be vaccinated and not require supplemental oxygen during acute illness.

Among the 162 with known recovery status at 9 months, 41% reported persistent symptoms, 73% of whom had been hospitalized during acute illness. The median participant age was 54 years (range, 24–94 years); approximately half (56%) were female, non-Hispanic White (56%) and required hospitalization for their illness (47%). The majority of participants (78%) had at least 1 comorbidity at the time of diagnosis (Table 1). Among the 76 participants diagnosed during the circulation of a pre-Omicron variant, 62% had persistent symptoms beyond 9 months, compared with 23% diagnosed during the Omicron era. Similarly, of the 92 participants fully vaccinated or boosted prior to their COVID-19 diagnosis, 24% had persistent symptoms beyond 9 months, compared to 64% among those who were not fully vaccinated prior to infection.

**Table 1.** Demographic and Clinical Characteristics of Participants, Stratified by Recovery Status at 9 Months

Characteristic	Known Recovery Status 9 mo After Diagnosis			LTFU (n = 60)
	Recovered (n = 95)	Not Recovered (n = 67)	Overall (n = 162)	
Demographics				
Age, y	54 (24–94)	59 (27–82)	54 (24–94)	51 (26–93)
Female sex	53 (55.8)	38 (56.7)	91 (56.2)	19 (31.7)
Race/ethnicity				
Non-Hispanic White	59 (62.1)	32 (47.8)	91 (56.2)	18 (30.0)
Hispanic	7 (7.4)	14 (20.9)	21 (13.0)	23 (38.3)
Black/African American	22 (23.2)	16 (23.9)	38 (23.5)	11 (18.3)
Other/multiple	7 (7.4)	5 (7.5)	12 (7.4)	8 (13.3)
Participant baseline characteristics				
BMI, kg/m^2^				
Normal (<25)	25 (26.3)	9 (13.4)	34 (21.0)	11 (18.3)
Overweight (25–30)	28 (29.5)	22 (32.8)	50 (30.9)	22 (36.7)
Obese (>30)	41 (43.2)	35 (52.2)	76 (46.9)	24 (40.0)
Any comorbidities^a^	69 (72.6)	57 (85.1)	126 (77.8)	48 (80.0)
Immunocompromised	23 (24.2)	12 (17.9)	35 (21.6)	8 (13.3)
Diabetes	15 (15.8)	20 (29.9)	35 (21.6)	18 (30.0)
Hypertension	36 (37.9)	31 (46.3)	67 (41.4)	30 (50.0)
Smoking status				
Never	55 (57.9)	39 (58.2)	94 (58.0)	28 (46.7)
Current or former	38 (40.0)	28 (41.8)	66 (40.7)	26 (43.3)
COVID-19 illness characteristics				
Hospitalized for COVID-19	27 (28.4)	49 (73.1)	76 (46.9)	43 (71.7)
Highest supplemental oxygen				
None	73 (76.8)	23 (34.3)	96 (59.3)	22 (36.7)
Low-flow (≤20 L/min)	16 (16.8)	33 (49.3)	49 (30.2)	26 (43.3)
High-flow (≥30 L/min), NIV, or mechanical	6 (6.3)	11 (16.4)	17 (10.5)	12 (20.0)
Therapy received				
Antiviral (remdesivir or nirmatrelvir/ritonavir)	71 (74.7)	51 (76.1)	122 (75.3)	31 (51.7)
Immunomodulatory (corticosteroids, tocilizumab, baricitinib)	20 (21.1)	42 (62.7)	62 (38.3)	35 (58.3)
Vaccination status at diagnosis^c^				
Not vaccinated	8 (8.4)	6 (9.0)	14 (8.6)	21 (35.0)
Primary series incomplete	17 (17.9)	39 (58.2)	56 (34.6)	26 (43.3)
Primary series complete	11 (11.6)	6 (9.0)	17 (10.5)	4 (6.7)
Boosted	59 (62.1)	16 (23.9)	75 (46.3)	9 (15.0)
Presumed variant				
Pre-Omicron	29 (30.5)	47 (70.1)	76 (46.9)	49 (81.7)
Wild-type	16 (16.8)	33 (49.3)	49 (30.2)	31 (51.7)
Alpha	5 (5.3)	8 (11.9)	13 (8.0)	7 (11.7)
Delta	8 (8.4)	6 (9.0)	14 (8.6)	11 (18.3)
Omicron	66 (69.5)	20 (29.9)	86 (53.1)	11 (18.3)
Baseline laboratory measures^b^				
Detectable N Ag (*Quanterix*)	23 (24.2)	45 (67.2)	68 (42.0)	32 (53.3)
Detectable anti-N IgG (*Bio-Rad*)	48 (50.5)	37 (55.2)	85 (52.5)	41 (68.3)
Detectable anti-S IgG (*Genscript*)	82 (86.3)	59 (88.1)	141 (87.0)	45 (75.0)

Data are presented as No. (%) for categorical variables and median (range) for continuous variables. Two participants with known recovery status at 9 months were missing information on BMI and smoking status, and 1 participant was missing information on prior comorbidities and immunocompromising conditions. Among those who were lost to follow-up, baseline laboratory measures and smoking status were missing for 6 participants, 3 were missing BMI, and 2 were missing information on prior comorbidities and immunocompromising conditions.

Abbreviations: anti-N IgG, anti-nucleocapsid immunoglobulin G; anti-S IgG, anti-spike immunoglobulin G; BMI, body mass index; COVID-19, coronavirus disease 2019; LTFU, lost to follow-up; N Ag, nucleocapsid antigen; NIV, noninvasive ventilation.

^a^Binary composite of any of the following conditions: diabetes, hypertension, coronary heart disease or heart failure, chronic obstructive pulmonary disease, stroke, chronic kidney disease (stage 3 or greater), end-stage renal disease, or obesity (BMI ≥30 kg/m^2^).

^b^Detectable N Ag: >3 pg/mL (limit of quantification); detectable anti-N IgG: normalized signal-to-cutoff ratio ≥1.0; detectable anti-S IgG: binding inhibition ≥30%.

Participants enrolled as inpatients had acute blood samples collected earlier in the illness progression than their outpatient counterparts (median days from symptom onset, 7 vs 14, respectively; Supplementary Table 1). Participants had a median of 5 (range, 1–10) symptom assessments completed over follow-up. All but 20 participants had at least 1 assessment completed prior to their annual visit, 12 of whom subsequently reported persistent symptoms at their next visit (Supplementary Figure 1).

Among the participants with persistent symptoms beyond 9 months, there were 67 assessments completed at a visit when active symptoms were ongoing (Table 2). The most common systems involved were CNS/psychological (69%) and systemic (64%), and the most common individual symptoms included fatigue (51%), difficulty concentrating (48%), and memory loss/forgetfulness (37%) (Table 3). These symptomatic individuals also reported interference with physical activity (49%), work or job functions (31%), ADLs (22%), and social interactions (21%), with only 43% reporting they did not have any functional limitations due to their persistent symptoms.

**Table 2.** Recovery Status and Method of Assessment Among Symptomatic Participants Diagnosed With Coronavirus Disease 2019 Between 6 July 2020 and 31 December 2022, Where Recovery Status at 9 Months Is Used as the Analysis Set

Status	Cutoff Used to Assess Recovery Status			
	Acute (≥15 d)	≥3 mo	≥6 mo	≥9 mo
Not recovered at last assessment	…	0	1	2
Recovery status assessed	164	164	163	162
Cumulative recovered before cutoff	38 (32%)	79 (48%)	88 (54%)	95 (59%)
Symptoms assessed before cutoff	9	36	49	72
No assessment after cutoff while symptomatic	29	43	39	23
Cumulative not recovered at cutoff	127 (77%)	85 (52%)	75 (46%)	67 (41%)
Detailed symptom assessment occurred after cutoff	47	38	31	36
No assessment after cutoff while symptomatic	79	47	44	31

**Table 3.** Prevalence of Individual Symptoms and Functional Limitations Among Those With Detailed Symptom Assessment After Designated Recovery Threshold

Symptom	Not Recovered at Cutoff		
	≥3 mo (n = 85)	≥6 mo (n = 75)	≥9 mo (n = 67)
**Any upper respiratory**	**32 (37.6)**	**30 (40.0)**	**29 (43.3)**
Nasal congestion	20 (23.5)	16 (21.3)	15 (22.4)
Runny nose	15 (17.6)	12 (16.0)	11 (16.4)
Loss of smell	7 (8.2)	7 (9.3)	7 (10.4)
Loss of taste	10 (11.8)	10 (13.3)	8 (11.9)
Sore throat	5 (5.9)	4 (5.3)	4 (6.0)
**Any systemic**	**48 (56.5)**	**44 (58.7)**	**43 (64.2)**
Body aches	26 (30.6)	25 (33.3)	23 (34.3)
Fatigue	38 (44.7)	35 (46.7)	34 (50.7)
Feverish	5 (5.9)	4 (5.3)	3 (4.5)
Headache	25 (29.4)	23 (30.7)	22 (32.8)
**Any cardiopulmonary**	**36 (42.4)**	**34 (45.3)**	**31 (46.3)**
Difficulty breathing	26 (30.6)	24 (32.0)	22 (32.8)
Chest pain	9 (10.6)	8 (10.7)	7 (10.4)
Cough	15 (17.6)	10 (13.3)	10 (14.9)
**Any central nervous system/psychological**	**54 (63.5)**	**48 (64.0)**	**46 (68.7)**
Anxiety	26 (30.6)	23 (30.7)	23 (34.3)
Difficulty thinking/confusion	29 (34.1)	27 (36.0)	24 (35.8)
Depressed mood	22 (25.9)	20 (26.7)	19 (28.4)
Difficulty concentrating	38 (44.7)	36 (48.0)	32 (47.8)
Insomnia	23 (27.1)	22 (29.3)	21 (31.3)
Memory loss/forgetfulness	33 (38.8)	30 (40.0)	25 (37.3)
Excessive daytime sleepiness	26 (30.6)	21 (28.0)	16 (23.9)
**Any gastrointestinal**	**24 (28.2)**	**22 (29.3)**	**20 (29.9)**
Abdominal pain	11 (12.9)	10 (13.3)	9 (13.4)
Diarrhea	11 (12.9)	9 (12.0)	8 (0.11.9)
Nausea and/or vomiting	14 (16.5)	12 (16.0)	11 (16.4)
**Any dermatologic**	**19 (22.4)**	**18 (24.0)**	**16 (23.9)**
Hair loss	16 (18.8)	15 (20.0)	13 (19.4)
Skin rash	6 (7.1)	5 (6.7)	4 (6.0)
**Any functional limitations**	**44 (51.8)**	**43 (57.3)**	**38 (56.7)**
Activities of daily living	22 (25.9)	20 (26.7)	15 (22.4)
Work/job functions	26 (30.6)	25 (33.3)	21 (31.3)
Physical activity	37 (43.5)	36 (48.0)	33 (49.3)
Social interactions	18 (21.2)	15 (20.0)	14 (20.9)

Data are presented as No. (%).

In univariate analyses, measures of initial illness severity (hospitalization and oxygen requirement, immunomodulatory therapy during acute illness, vaccination status, variant, and N Ag level) were the strongest predictors of persistent symptoms beyond 9 months, as well as Hispanic ethnicity and diabetes (Table 4). After adjustment for demographics, comorbidities, and disease severity, a detectable N Ag level and requiring supplemental oxygen during acute illness remained significantly associated with persistent long COVID symptoms at 9 months (adjusted ORs, 3.0 [95% CI, 1.1–8.0] and 3.6 [95% CI, 1.2–11] respectively; Table 4). These findings were consistent when models were repeated using 3- and 6-month timepoints (Supplementary Table 2).

**Table 4.** Association of Baseline and Illness Characteristics With Odds of Persistent Symptoms at 9 Months

Characteristic (n = 162)	Model 1 OR (95% CI)	Model 2 aOR (95% CI)	Model 3 aOR (95% CI)	Model 4 aOR (95% CI)
Demographics				
Age (per 10 y)	1.2 (1.0–1.5)	1.2 (.9–1.5)	1.1 (.9–1.5)	1.1 (.9–1.5)
Female sex (vs male)	1.0 (.6–2.0)	1.2 (.6–2.4)	1.8 (.8–3.8)	1.9 (.9–4.2)
Race/ethnicity				
Non-Hispanic White	Ref	Ref	Ref	Ref
Hispanic	3.7 (1.4–10)*	5.4 (1.8–16)**	1.8 (.5–6.1)	1.9 (.5–6.2)
Black/African American	1.3 (.6–2.9)	1.3 (.6–2.9)	0.5 (.2–1.4)	0.6 (.2–1.6)
Other/multiple	1.3 (.4–4.5)	1.9 (.5–7.0)	1.2 (.3–4.8)	1.2 (.3–5.1)
Participant baseline characteristics				
Body mass index, kg/m^2^				
Normal (<25)	Ref	Ref	Ref	Ref
Overweight (25–30)	2.2 (.8–5.6)	1.6 (.6–4.6)	1.6 (.5–4.8)	1.5 (.5–4.6)
Obese (>30)	2.4 (1.0–5.7)	2.0 (.7–5.6)	1.2 (.4–3.7)	1.1 (.3–3.3)
Any comorbidities	2.1 (.9–4.7)	2.3 (.9–5.9)	2.3 (.8–6.4)	2.2 (.8–6.1)
Immunocompromised	0.7 (.3–1.5)	0.5 (.2–1.3)	0.6 (.2–1.5)	0.5 (.2–1.4)
Diabetes	2.3 (1.1–4.9)*	2.0 (.8–4.7)	1.7 (.7–4.4)	1.6 (.6–4.3)
Hypertension	1.4 (.7–2.7)	0.8 (.4–1.9)	0.9 (.4–2.1)	0.8 (.3–2.0)
Ever smoker (vs never)	1.0 (.5–2.0)	1.1 (.5–2.2)	1.0 (.5–2.0)	1.0 (.4–2.1)
COVID-19 illness characteristics				
Hospitalized for COVID-19	6.9 (3.4–14)***	9.1 (3.7–23)***	4.9 (1.1–23)*	2.4 (.4–16)
Any supplemental oxygen (vs none)	6.3 (3.2–13)***	7.7 (3.2–19)***	7.7 (3.2–19)***	3.6 (1.2–11)*
Therapy received				
Antiviral (remdesivir or nirmatrelvir/ritonavir)	1.1 (.5–2.2)	0.9 (.4–2.0)	0.6 (.2–1.4)	0.7 (.3–1.7)
Immunomodulatory (corticosteroids, tocilizumab, baricitinib)	6.3 (3.1–13)***	5.7 (2.6–12)***	1.7 (.4–6.8)	1.3 (.3–5.6)
Incomplete vaccination (vs primary series complete and/or boosted)	5.7 (2.9–11)***	7.5 (3.2–18)***	3.6 (1.2–11)*	2.5 (.7–8.4)
Pre-Omicron variant (vs Omicron)	5.3 (2.7–11)***	5.9 (2.5–14)***	2.5 (.9–7.2)	1.7 (.5–5.4)
Baseline laboratory measures^a^				
Detectable N Ag (*Quanterix*)	6.4 (3.2–13)***	6.2 (2.9–13)***	3.0 (1.1–8.0)*	3.0 (1.1–8.0)*
Detectable anti-N IgG (*Bio-Rad*)	1.3 (.7–2.4)	1.2 (.6–2.3)	0.8 (.4–1.7)	0.8 (.4–1.7)
Detectable anti-S IgG (*Genscript*)	0.9 (.4–2.2)	0.8 (.3–2.2)	1.1 (.3–3.1)	1.4 (.5–4.1)

Abbreviations: anti-N IgG, anti-nucleocapsid immunoglobulin G; anti-S IgG, anti-spike immunoglobulin G; aOR, adjusted odds ratio; CI, confidence interval; COVID-19, coronavirus disease 2019; N Ag, nucleocapsid antigen; OR, odds ratio; Ref, Referent.

^a^Detectable N Ag: >3 pg/mL (limit of quantification); detectable anti-N IgG: normalized signal-to-cutoff ratio ≥1.0; detectable anti-S IgG: binding inhibition ≥30%.

^*^ *P* < .05.

^**^ *P* < .01.

***P < .001.

## DISCUSSION

In this prospective cohort study of patients with acute COVID-19 illness during both pre- and Omicron eras (2020–2022), 41% of participants had persistent symptoms beyond 9 months of initial infection, 73% of whom had been hospitalized during their initial infection. We found that disease severity and viral burden during acute illness are independently associated with greater risk of long COVID, even after adjusting for baseline demographic characteristics and comorbid conditions. Among those not recovered, approximately two-thirds reported at least 1 central nervous/psychological (eg, difficulty concentrating, forgetfulness) or systemic symptom (eg, fatigue), and a majority reported having 1 or more functional limitations as a result of those persistent symptoms.

The prevalence of long COVID has been estimated to occur in anywhere from 7% to 93% infected individuals [17–21]. Several meta-analyses of >100 studies found pooled estimates of long COVID prevalence around 45%, though most of the included studies were also categorized as having a high risk of bias due to study design, follow-up length, sample source, and outcome definitions [22, 23]. This heterogeneity could be explained by variable study populations. For example, studies that exclusively reported on patients who had severe acute illness, required hospitalization, or were infected earlier in the pandemic (2020–2021) estimated a higher prevalence of long COVID than studies of individuals recruited in outpatient or community-based settings, or those infected with an Omicron lineage of the virus. We found the risk of long COVID varied by variant and vaccination status, consistent with Xie at al, who recently found that the temporal reduction in long COVID incidence over time was attributable to both vaccine- (69% attributable risk) and era-related effects (30% attributable risk) [24].

Disease severity has been demonstrated as a risk factor for long COVID, but the underlying mechanisms remain poorly understood [8]. There are likely many factors at play, but hyperinflammation from increased acute cytokine production has been hypothesized as a key driver [25–27]. Plasma N antigen level has been previously identified as a biomarker for viral burden that is predictive of disease prognosis during acute illness [15]. We also report an independent association between detectable acute N antigen levels and long COVID, emphasizing the potential importance of acute viral burden in long COVID pathogenesis. Swank et al found sustained and fluctuating concentrations of the S1 subunit of the spike protein, full-length spike, and N antigen in long COVID patients. As long as a year after acute illness, they found detectable spike protein in 60% of patients reporting long COVID but not in controls [28]. Viral persistence in blood and solid tissues and long COVID are inextricably linked, but the underlying immunologic mechanism by which impaired viral clearance gives rise to persistent (long COVID) symptoms—perhaps through the promotion of a proinflammatory state—has yet to be fully elucidated [29, 30]. In large-scale studies summarizing the burden of long COVID, shortness of breath, fatigue, and cognitive dysfunction have been the most frequently reported symptoms [31, 32], in line with our finding that CNS and psychological systems were the most commonly involved. Our estimates of the impact of long COVID on quality of life are consistent with others reported in the literature: Vélez-Santamaría et al found that 45% of participants with long COVID reported decreased physical activity and 54% reported an overall reduced quality of life [33]. Of long COVID patients presenting to a post-COVID rehabilitation program in 2020 (on average, 3 months after acute infection), a third had difficulties with ADLs and 84% reported other functional limitations including work tasks, driving, and exercise [34]. Our study expands on these findings by describing and quantifying long COVID morbidity (ADLs, work and job functions, physical activity, and social interactions) over time, across both hospitalized and nonhospitalized, and pre-Omicron and Omicron patients.

Our study began long before the NASEM's definition of long COVID was proposed [7], but the symptom assessment we developed evaluated the majority of the common symptoms listed in the current definition—cough, fatigue, difficulty concentrating, memory changes, headache, sleep disturbances, loss of taste or smell, diarrhea, and mood disorders. When defining long COVID, we assumed that symptom resolution was permanent, that is, once recovery was noted, long COVID symptoms could not redevelop. However, NASEM's definition acknowledges that the presentation of long COVID may be delayed, relapsing, remitting, or progressive.

There are several limitations to be noted. First, approximately 25% of participants were lost to follow-up prior to ascertainment of their recovery status at the beginning of the annual visit window (9 months). Demographic and clinical information show that those lost to follow-up in our cohort were more likely to be Hispanic, have comorbidities such as diabetes and hypertension, be unvaccinated, and had more severe acute illness requiring hospitalization and supplemental oxygen. Thus, our findings are likely conservative and underestimate the true prevalence of long COVID. Second, we assumed that symptoms, functional limitations, and recovery status were present from acute illness through the time they were first reported to us. This is subject to recall bias, particularly for the subset of participants whose first symptom assessment occurred 6 months or more after acute illness. Misclassification bias may also impact our findings, as we were unable to account for temporal fluctuations in long COVID disease state. Finally, as with any observational study, our findings may be influenced by residual confounding.

Few long COVID studies have prospectively followed individuals from acute illness and instead rely on either cross-sectional/retrospective symptom ascertainment or *International Classification of Diseases, Tenth Revision* coding. As a longitudinal cohort, we were able to document fluctuations in severity of individual symptoms over time, across several predominant strains of SARS-CoV-2, and within a rapidly changing vaccine climate. Our findings reinforce that long COVID has been very common during the initial years of the pandemic, often characterized by fatigue and neurocognitive symptoms that are associated with long-term physical and functional limitations. The strong association with initial disease severity suggests the prevalence and impact of long COVID may decrease as acute illnesses have become milder over time. However, a subset of contemporary patients still had a high viral burden coupled with extended periods of viral replication during acute illness, even after vaccination, so implications for long COVID in the current era remain unclear [24]. These findings emphasize the importance of continuing research to characterize long COVID risk as the spectrum of acute illness changes along with viral evolution of SARS-CoV-2.

## Supplementary Data

Supplementary materials are available at *Open Forum Infectious Diseases* online. Consisting of data provided by the authors to benefit the reader, the posted materials are not copyedited and are the sole responsibility of the authors, so questions or comments should be addressed to the corresponding author.

## Supplementary Material

ofaf048_Supplementary_Data

## Notes

***Acknowledgments.*** We thank Jonathan Klaphake and Kelly Garcia-Meyers for their guidance and expertise in creating and sustaining our study cohort. We also thank all of our study participants for their ongoing and invaluable contributions to advancing the scientific understanding of COVID-19.

***Data availability.*** Data are not publicly available.

***Financial support*.** This work was supported in part by internal Hennepin Healthcare Research Institute nondiscretionary funding available to J. V. B. J. V. B. and C. S. were supported by a grant (R01HL158756-03) from the National Heart, Lung, and Blood Institute of the National Institutes of Health (NIH). The work of I. S. was supported by the Intramural Research Program of the National Institute of Allergy and Infectious Diseases, NIH.

## References

[1] Davis HE, Assaf GS, McCorkell L, et al Characterizing long COVID in an international cohort: 7 months of symptoms and their impact. eClinicalMedicine 2021; 38:101019. 34308300 10.1016/j.eclinm.2021.101019 PMC8280690

[2] Centers for Disease Control and Prevention . Clinical overview of long COVID. 2024. Available at: https://www.cdc.gov/covid/hcp/clinical-overview/index.html. Accessed 19 August 2024.

[3] National Institute for Health and Care Excellence. Overview COVID-19 rapid guideline: managing COVID-19 guidance. 2021. Available at: https://www.nice.org.uk/guidance/ng191. Accessed 19 August 2024. 34181371

[4] Soriano JB, Murthy S, Marshall JC, Relan P, Diaz JV. A clinical case definition of post-COVID-19 condition by a Delphi consensus. Lancet Infect Dis 2022; 22:e102–7. 34951953 10.1016/S1473-3099(21)00703-9 PMC8691845

[5] Thaweethai T, Jolley SE, Karlson EW, et al Development of a definition of postacute sequelae of SARS-CoV-2 infection. JAMA 2023; 329:1934–46. 37278994 10.1001/jama.2023.8823 PMC10214179

[6] Ely EW, Brown LM, Fineberg HV. Long Covid defined. N Engl J Med 2024; 391:1746–53. 39083764 10.1056/NEJMsb2408466 PMC11687645

[7] Fineberg HV, Brown L, Worku T, Goldowitz, I eds. A long COVID definition: a chronic, systemic disease state with profound consequences. Washington, DC: National Academies Press, 2024. 39110819

[8] Altmann DM, Whettlock EM, Liu S, Arachchillage DJ, Boyton RJ. The immunology of long COVID. Nat Rev Immunol 2023; 23:618–34. 37433988 10.1038/s41577-023-00904-7

[9] Ayoubkhani D, Bosworth ML, King S, et al Risk of long COVID in people infected with severe acute respiratory syndrome coronavirus 2 after 2 doses of a coronavirus disease 2019 vaccine: community-based, matched cohort study. Open Forum Infect Dis 2022; 9:ofac464. 36168555 10.1093/ofid/ofac464 PMC9494414

[10] Zhao Y, Shi L, Jiang Z, et al The phenotype and prediction of long-term physical, mental and cognitive COVID-19 sequelae 20 months after recovery, a community-based cohort study in China. Mol Psychiatry 2023; 28:1793–801. 36690792 10.1038/s41380-023-01951-1 PMC9869317

[11] Rahmati M, Udeh R, Yon DK, et al A systematic review and meta-analysis of long-term sequelae of COVID-19 2-year after SARS-CoV-2 infection: a call to action for neurological, physical, and psychological sciences. J Med Virol 2023; 95:e28852. 37288652 10.1002/jmv.28852

[12] Harris PA, Taylor R, Minor BL, et al The REDCap consortium: building an international community of software platform partners. J Biomed Inform 2019; 95:103208. 31078660 10.1016/j.jbi.2019.103208 PMC7254481

[13] Harris PA, Taylor R, Thielke R, Payne J, Gonzalez N, Conde JG. Research electronic data capture (REDCap)—a metadata-driven methodology and workflow process for providing translational research informatics support. J Biomed Inform 2009; 42:377–81. 18929686 10.1016/j.jbi.2008.08.010 PMC2700030

[14] Hodcroft EB . CoVariants: SARS-CoV-2 mutations and variants of interest. 2021. Available at: https://covariants.org/. Accessed 26 April 2024.

[15] ACTIV-3/TICO Study Group . The association of baseline plasma SARS-CoV-2 nucleocapsid antigen level and outcomes in patients hospitalized with COVID-19. Ann Intern Med 2022; 175:1401–10. 36037469 10.7326/M22-0924 PMC9447373

[16] Tan CW, Chia WN, Qin X, et al A SARS-CoV-2 surrogate virus neutralization test based on antibody-mediated blockage of ACE2-spike protein-protein interaction. Nat Biotechnol 2020; 38:1073–8. 32704169 10.1038/s41587-020-0631-z

[17] Sigfrid L, Drake TM, Pauley E, et al Long Covid in adults discharged from UK hospitals after Covid-19: a prospective, multicentre cohort study using the ISARIC WHO clinical characterisation protocol. Lancet Reg Health Eur 2021; 8:100186. 34386785 10.1016/j.lanepe.2021.100186 PMC8343377

[18] Yan B, Song L, Guo J, Wang Y, Peng L, Li D. Association between clinical characteristics and short-term outcomes in adult male COVID-19 patients with mild clinical symptoms: a single-center observational study. Front Med (Lausanne) 2021; 7:571396. 33469542 10.3389/fmed.2020.571396 PMC7813813

[19] Office for National Statistics . Prevalence of ongoing symptoms following coronavirus (COVID-19) infection in the UK: 30 March 2023. 2023. Available at: https://www.ons.gov.uk/peoplepopulationandcommunity/healthandsocialcare/conditionsanddiseases/bulletins/prevalenceofongoingsymptomsfollowingcoronaviruscovid19infectionintheuk/30march2023. Accessed 1 May 2024.

[20] National Center for Health Statistics . Long COVID. 2024. Available at: https://www.cdc.gov/nchs/covid19/pulse/long-covid.htm. Accessed 18 April 2024.

[21] Davis HE, McCorkell L, Vogel JM, Topol EJ. Long COVID: major findings, mechanisms and recommendations. Nat Rev Microbiol 2023; 21:133–46. 36639608 10.1038/s41579-022-00846-2 PMC9839201

[22] Di Gennaro F, Belati A, Tulone O, et al Incidence of long COVID-19 in people with previous SARS-Cov2 infection: a systematic review and meta-analysis of 120,970 patients. Intern Emerg Med 2023; 18:1573–81. 36449260 10.1007/s11739-022-03164-w PMC9709360

[23] Woodrow M, Carey C, Ziauddeen N, et al Systematic review of the prevalence of long COVID. Open Forum Infect Dis 2023; 10:ofad233. 37404951 10.1093/ofid/ofad233 PMC10316694

[24] Xie Y, Choi T, Al-Aly Z. Postacute sequelae of SARS-CoV-2 infection in the pre-Delta, Delta, and Omicron eras. N Engl J Med 2024; 391:515–25. 39018527 10.1056/NEJMoa2403211 PMC11687648

[25] Mehandru S, Merad M. Pathological sequelae of long-haul COVID. Nat Immunol 2022; 23:194–202. 35105985 10.1038/s41590-021-01104-y PMC9127978

[26] Hadjadj J, Yatim N, Barnabei L, et al Impaired type I interferon activity and inflammatory responses in severe COVID-19 patients. Science 2020; 369:718–24. 32661059 10.1126/science.abc6027 PMC7402632

[27] Vabret N, Britton GJ, Gruber C, et al Immunology of COVID-19: current state of the science. Immunity 2020; 52:910–41. 32505227 10.1016/j.immuni.2020.05.002 PMC7200337

[28] Swank Z, Senussi Y, Manickas-Hill Z, et al Persistent circulating severe acute respiratory syndrome coronavirus 2 spike is associated with post-acute coronavirus disease 2019 sequelae. Clin Infect Dis 2022; 76:e487–90. 10.1093/cid/ciac722 PMC10169416 36052466

[29] Zuo W, He D, Liang C, et al The persistence of SARS-CoV-2 in tissues and its association with long COVID symptoms: a cross-sectional cohort study in China. Lancet Infect Dis 2024; 24:845–55. 38663423 10.1016/S1473-3099(24)00171-3

[30] Buonsenso D, Tantisira KG. Long COVID and SARS-CoV-2 persistence: new answers, more questions. Lancet Infect Dis 2024; 24:796–8. 38663424 10.1016/S1473-3099(24)00216-0

[31] Peghin M, Palese A, Venturini M, et al Post-COVID-19 symptoms 6 months after acute infection among hospitalized and non-hospitalized patients. Clin Microbiol Infect 2021; 27:1507–13. 34111579 10.1016/j.cmi.2021.05.033 PMC8180450

[32] Alkodaymi MS, Omrani OA, Fawzy NA, et al Prevalence of post-acute COVID-19 syndrome symptoms at different follow-up periods: a systematic review and meta-analysis. Clin Microbiol Infect 2022; 28:657–66. 35124265 10.1016/j.cmi.2022.01.014 PMC8812092

[33] Vélez-Santamaría R, Fernández-Solana J, Méndez-López F, et al Functionality, physical activity, fatigue and quality of life in patients with acute COVID-19 and long COVID infection. Sci Rep 2023; 13:19907. 37963962 10.1038/s41598-023-47218-1 PMC10645778

[34] Vanichkachorn G, Newcomb R, Cowl CT, et al Post–COVID-19 syndrome (long haul syndrome): description of a multidisciplinary clinic at Mayo Clinic and characteristics of the initial patient cohort. Mayo Clin Proc 2021; 96:1782–91. 34218857 10.1016/j.mayocp.2021.04.024 PMC8112396

