# Supplementary material for: Viral Burden and Illness Severity During Acute SARS-CoV-2 Infection Predict Persistent Long COVID Symptoms
Source: Open Forum Infect Dis. 2025 Jan 30;12(2):ofaf048. doi: 10.1093/ofid/ofaf048 (PMC11800476; doi:10.1093/ofid/ofaf048)

Table of Contents

[Table S1. Distribution of sample collection by hospitalization status (N=162). 2](#_Toc187155655)

[Table S2. Unadjusted and fully adjusted odds ratios for not recovered at 3-, 6-, and 9-months post-diagnosis. 3](#_Toc187155656)

[Figure S1. Distribution of symptom assessments completed by unique participants with known recovery status at nine months. Symptom assessments were discontinued after complete recovery was first noted. 4](#_Toc187155657)

[Figure S2A-C. Distribution of acute lab measures by collection date and hospitalization status (N=162). 5](#_Toc187155658)

[A) *Anti-Nucleocapsid Immunoglobulin (Anti-N IgG [Bio-Rad]) where dashed line represents ≥1.0 S/Co ratio (detectable).* 5](#_Toc187155659)

[B) *Anti-Spike Neutralizing Immunoglobulin (Anti-S IgG [Genscript]) where dashed line represents detectable binding inhibition (≥30%).* 6](#_Toc187155660)

[C) *Nucleocapsid antigen (N Antigen [Quanterix]) where dashed line represents lower limit of quantification (3 pg/mL).* 7](#_Toc187155661)

# **Table S1.** Distribution of sample collection by hospitalization status (N=162).

|  | **Days Elapsed from Diagnosis to Sample Collection** | | | | |
| --- | --- | --- | --- | --- | --- |
|  | **Minimum** | **25^th^ Percentile** | **Median** | **75^th^ Percentile** | **Maximum** |
| **Overall** | 0 | 3 | 8 | 12 | 15 |
| **Inpatient** | 0 | 2 | 3 | 6 | 14 |
| **Outpatient** | 5 | 10 | 11 | 13 | 15 |
|  | **Days Elapsed from Symptom Onset to Sample Collection** | | | | |
|  | **Minimum** | **25^th^ Percentile** | **Median** | **75^th^ Percentile** | **Maximum** |
| **Overall** | 0 | 7 | 10 | 14 | 39 |
| **Inpatient** | 0 | 4.5 | 7 | 10.5 | 39 |
| **Outpatient** | 5 | 10 | 14 | 15 | 22 |

# **Table S2.** Unadjusted and fully adjusted odds ratios for not recovered at 3-, 6-, and 9-months post-diagnosis.

|  | **3 months [N = 85/164 (52%)]** | | **6 months [N =75/163 (46%)]** | | **9 months [N = 67/162 (41%)]** | |
| --- | --- | --- | --- | --- | --- | --- |
|  | **OR (95% CI)** | **aOR  (95% CI)** | **OR (95% CI)** | **aOR (95% CI)** | **OR (95% CI)** | **aOR (95% CI)** |
| **Demographics** |  |  |  |  |  |  |
| Age (per 10 years) | 1.1 (0.9-1.3) | 1.0 (0.8-1.3) | 1.1 (0.9-1.4) | 1.1 (0.8-1.4) | 1.2 (1.0-1.5) | 1.1 (0.9-1.5) |
| Female Birth Sex (vs. Male) | 1.1 (0.6-2.1) | 1.9 (0.9-4.0) | 1.1 (0.6-2.1) | 2.2 (1.0-4.8) | 1.0 (0.6-2.0) | 1.9 (0.9-4.3) |
| Race/Ethnicity |  |  |  |  |  |  |
| Non-Hispanic White | Ref. | Ref. | Ref. | Ref. | Ref. | Ref. |
| Hispanic | **3.1 (1.1-8.7)*** | 1.8 (0.5-6.2) | **4.1 (1.4-11)**** | 2.2 (0.6-8.0) | **3.7 (1.4-10)*** | 1.8 (0.5-6.1) |
| Black/African American | 1.8 (0.8-3.8) | 1.0 (0.4-2.5) | 1.6 (0.8-3.5) | 0.7 (0.3-2.0) | 1.3 (0.6-2.9) | 0.6 (0.2-1.6) |
| Other/Multiple | 1.2 (0.4-4.1) | 1.3 (0.3-5.0) | 1.6 (0.5-5.4) | 1.7 (0.4-7.2) | 1.3 (0.4-4.5) | 1.2 (0.3-5.0) |
| **Participant Baseline Characteristics** |  |  |  |  |  |  |
| Body Mass Index (kg/m^2^) |  |  |  |  |  |  |
| Normal (<25) | Ref. | Ref. | Ref. | Ref. | Ref. | Ref. |
| Overweight (25-30) | 2.2 (0.9-5.3) | 1.7 (0.6-4.9) | 2.0 (0.8-5.2) | 1.4 (0.4-4.2) | 2.2 (0.8-5.6) | 1.5 (0.5-4.7) |
| Obese (>30) | **2.5 (1.1-5.8)*** | 1.1 (0.4-3.2) | **2.7 (1.2-6.5)*** | 1.2 (0.4-3.7) | 2.4 (1.0-5.7) | 1.1 (0.3-3.4) |
| Any Comorbidities | **2.5 (1.2-5.6)*** | **2.8 (1.1-7.2)*** | **2.6 (1.2-5.9)*** | **3.3 (1.1-9.5)*** | 2.1 (0.9-4.7) | 2.2 (0.8-6.1) |
| Immunocompromised | 1.1 (0.5-2.2) | 0.9 (0.4-2.2) | 0.9 (0.4-1.9) | 0.8 (0.3-2.0) | 0.7 (0.3-1.5) | 0.5 (0.2-1.4) |
| Diabetes | **2.2 (1.0-4.8)*** | 1.4 (0.6-3.6) | **2.6 (1.2-5.5)*** | 1.8 (0.7-4.7) | **2.3 (1.1-4.9)*** | 1.7 (0.6-4.4) |
| Hypertension | 1.5 (0.8-2.7) | 0.9 (0.4-2.0) | 1.6 (0.9-3.0) | 1.0 (0.4-2.3) | 1.4 (0.8-2.7) | 0.8 (0.4-2.0) |
| Ever Smoker (vs. Never) | 1.1 (0.6-2.0) | 1.0 (0.5-2.1) | 0.9 (0.4-1.9) | 0.9 (0.4-1.8) | 1.0 (0.5-2.0) | 1.0 (0.5-2.1) |
| **COVID-19 Illness Characteristics** |  |  |  |  |  |  |
| Hospitalized for COVID-19 | **4.0 (2.1-7.8)***** | 0.7 (0.1-4.9) | **6.0 (3.1-12)***** | 1.4 (0.2-9.4) | **6.9 (3.4-14)***** | 2.5 (0.4-17) |
| Any Supplemental Oxygen (vs. None) | **4.4 (2.2-8.7)***** | 2.6 (0.9-7.5) | **6.1 (3.1-12)***** | **3.5 (1.2-10)*** | **6.3 (3.2-13)***** | **3.8 (1.3-11)*** |
| Incomplete vaccination  (vs. primary series complete and/or boosted) | **3.8 (1.9-7.3)***** | 1.5 (0.5-4.9) | **5.3(2.7-10)***** | 2.2 (0.6-7.2) | **5.7 (2.9-11)***** | 2.5 (0.7-8.4) |
| Pre-Omicron Variant (vs. Omicron) | **3.2 (1.7-6.2)***** | 0.8 (0.2-2.5) | **4.8 (2.5-9.3)***** | 1.2 (0.4-3.9) | **5.3 (2.7-11)***** | 1.7 (0.5-5.5) |
| **Baseline Lab Measures** |  |  |  |  |  |  |
| Detectable N Ag (*Quanterix*) | **4.0 (2.1-7.8)***** | 2.1 (0.8-5.5) | **5.7 (2.9-11)***** | **2.7 (1.1-7.2)*** | **6.1 (3.0-12)***** | **2.9 (1.1-7.5)*** |
| Detectable Anti-N IgG (*Bio-Rad*) | 1.3 (0.7-2.3) | 1.0 (0.5-1.9) | 1.3 (0.7-2.5) | 0.9 (0.4-1.9) | 1.2 (0.6-2.3) | 0.7 (0.3-1.6) |
| Detectable Anti-S IgG (*Genscript*) | 0.6 (0.2-1.6) | 0.9 (0.3-2.5) | 0.7 (0.3-1.9) | 1.1 (0.4-3.2) | 1.2 (0.5-3.0) | 1.9 (0.6-5.7) |

N Ag = nucleocapsid antigen; Anti-N = Anti-nucleocapsid; Anti-S = Anti-spike; IgG = immunoglobulin. * p-value <0.05; ** p-value <0.01; *** p-value <0.001. Detectable N Ag: >3 pg/mL (limit of quantification); Detectable Anti-N IgG: normalized signal-to-cutoff ratio ≥1.0; Detectable Anti-S IgG: binding inhibition ≥30%.

# **Figure S1.** Distribution of symptom assessments completed by unique participants with known recovery status at nine months. Symptom assessments were discontinued after complete recovery was first noted.

**
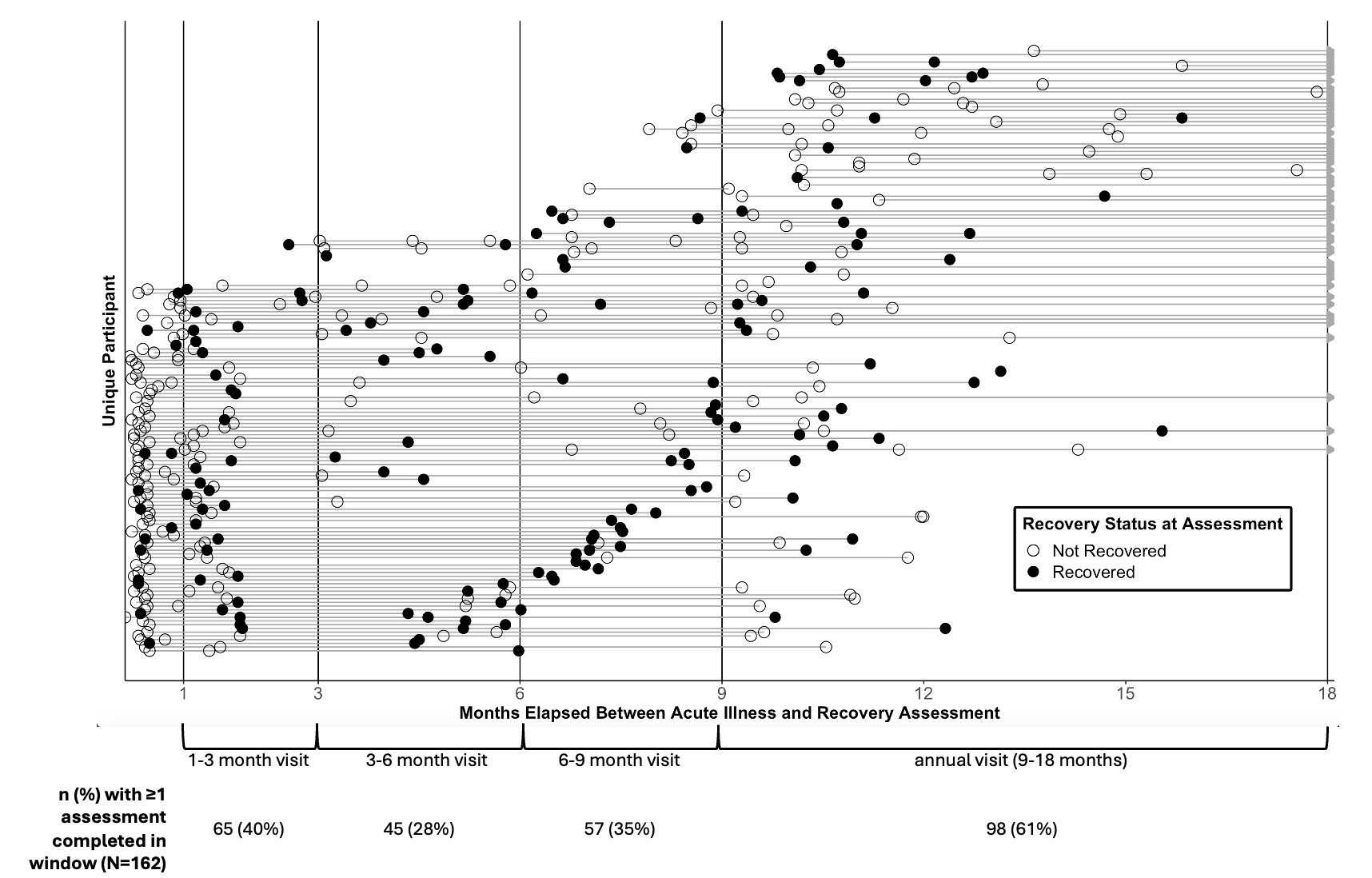
**

# **Figure S2A-C**. Distribution of acute lab measures by collection date and hospitalization status (N=162).

## Anti-Nucleocapsid Immunoglobulin (Anti-N IgG [*Bio-Rad]*) where dashed line represents ≥1.0 S/Co ratio (detectable).


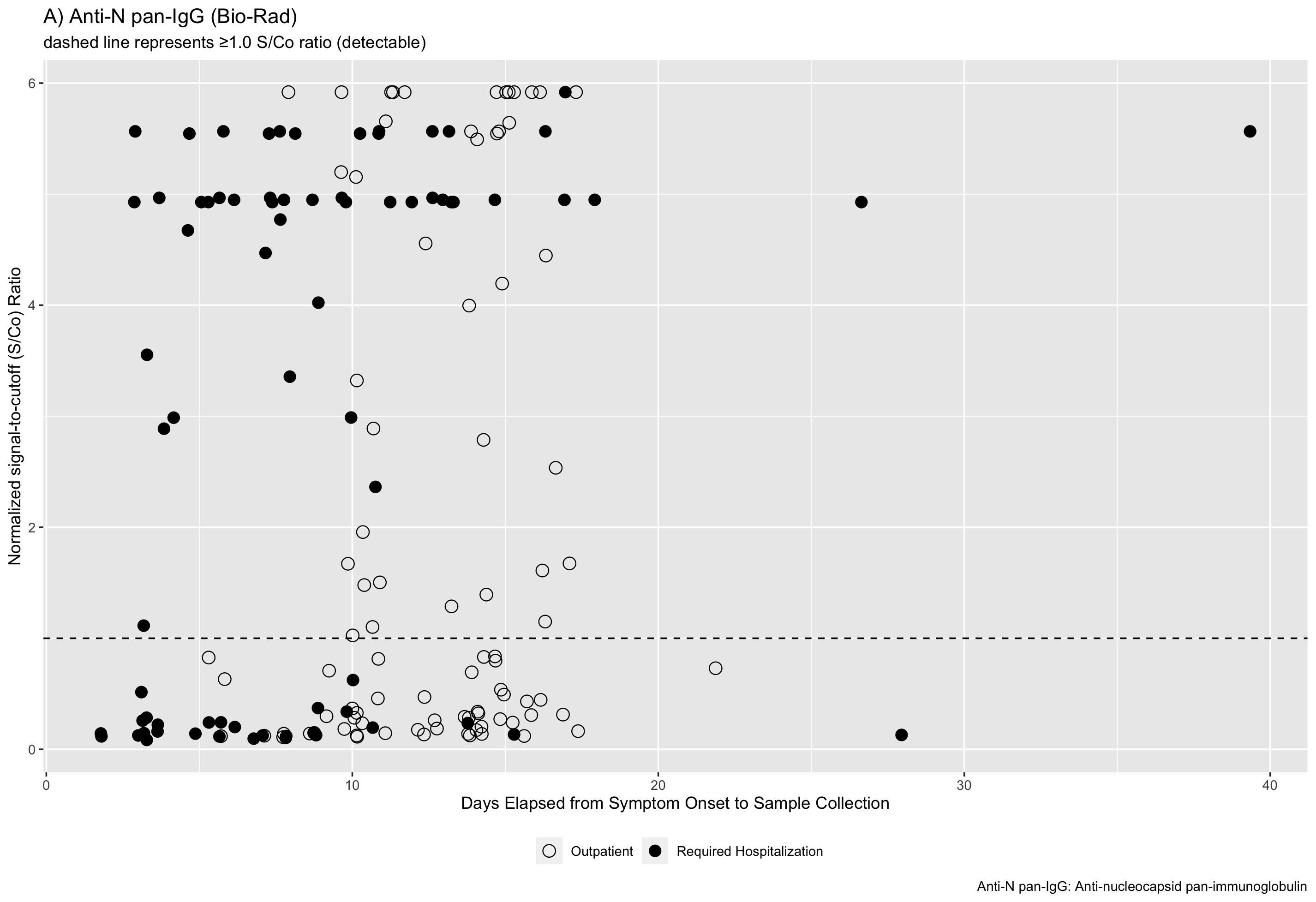


## Anti-Spike Neutralizing Immunoglobulin (Anti-S IgG [*Genscript*]) where dashed line represents detectable binding inhibition (≥30%).


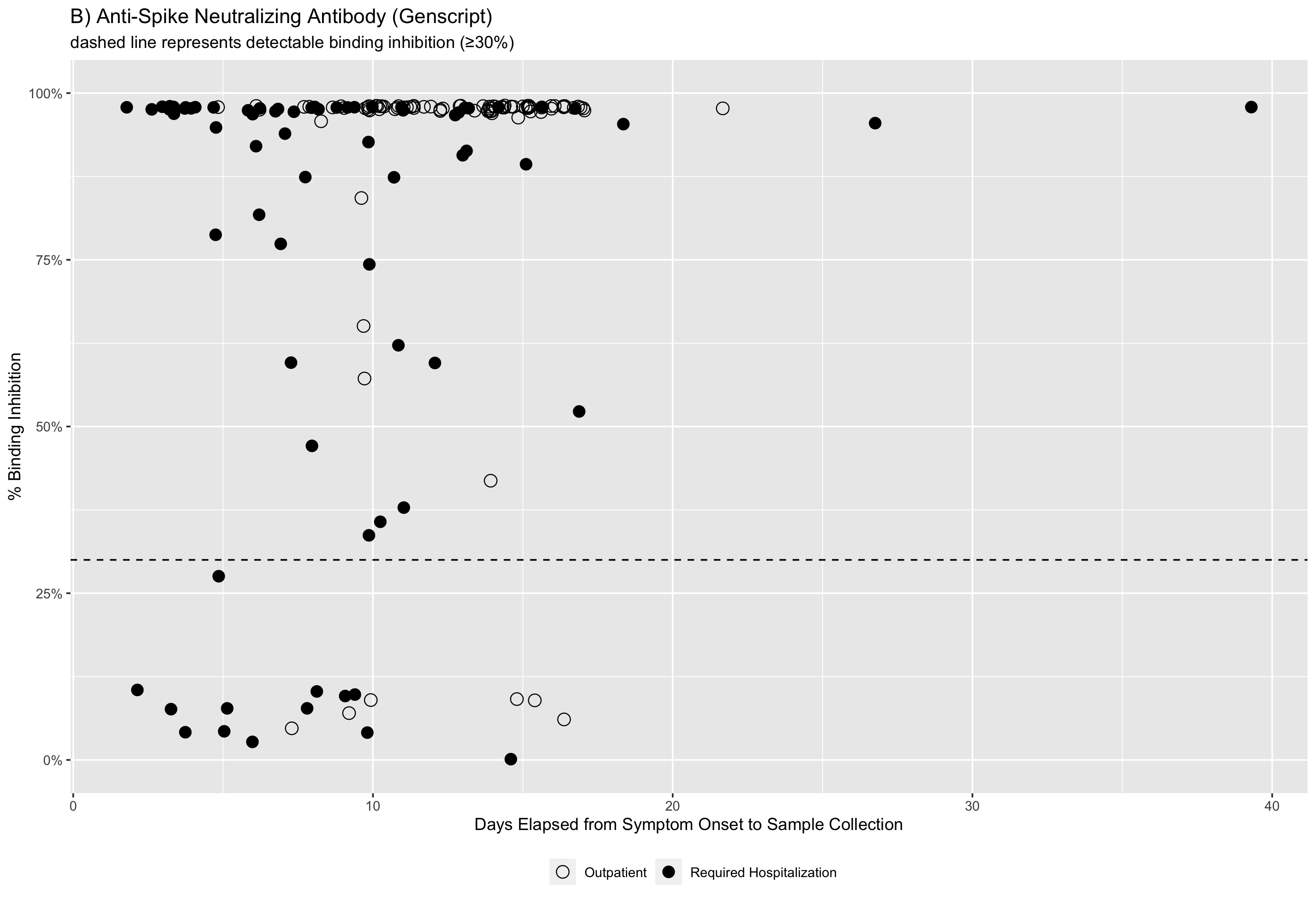


## Nucleocapsid antigen (N Antigen [*Quanterix*]) where dashed line represents lower limit of quantification (3 pg/mL).


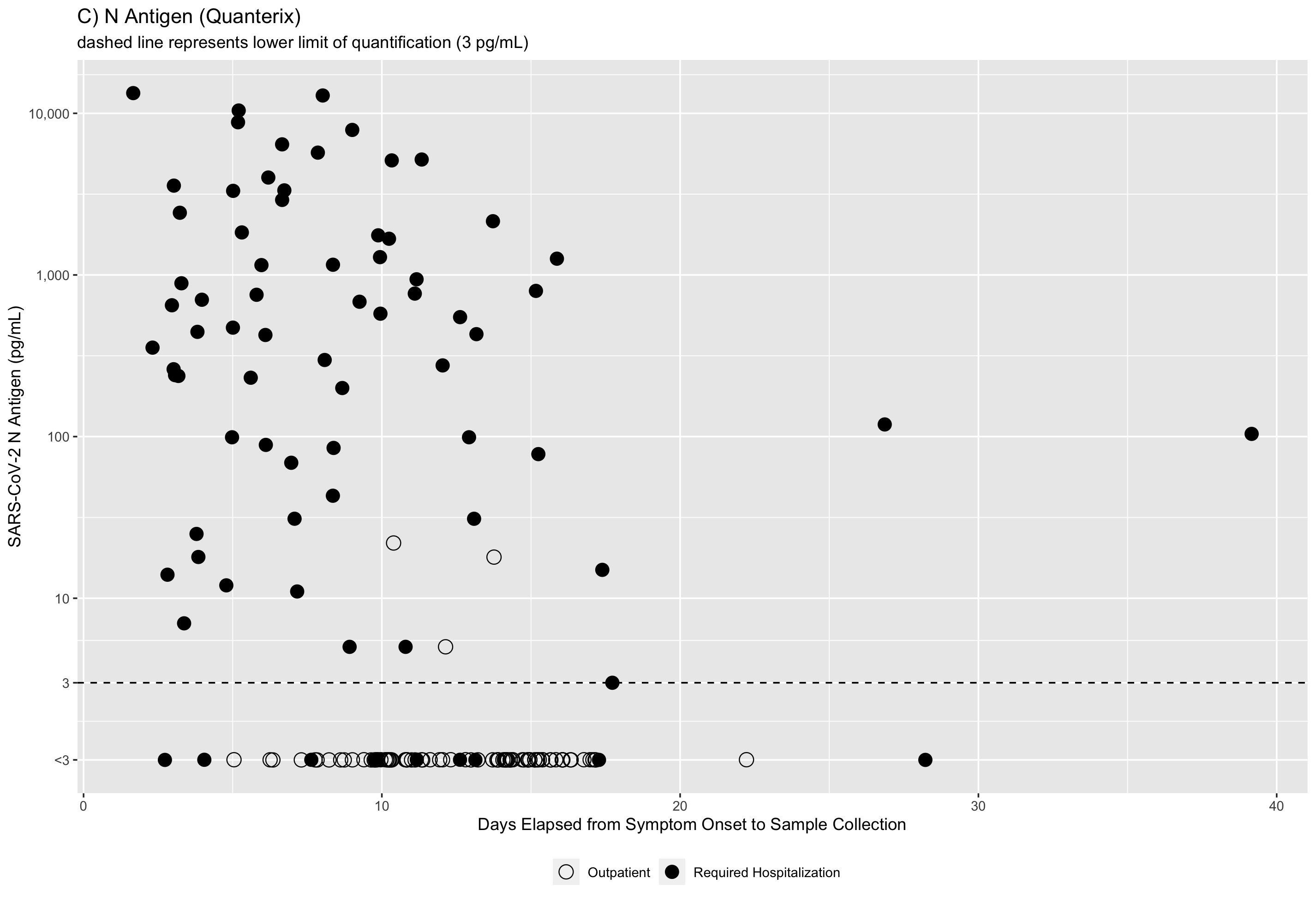

Supplement: ofaf048_Supplementary_Data [file ofaf048_supplementary_data.zip › Long COVID_SuppMaterials_010725.docx]
