# Supplementary material for: Viral Burden and Illness Severity During Acute SARS-CoV-2 Infection Predict Persistent Long COVID Symptoms
Source: Open Forum Infect Dis. 2025 Jan 30;12(2):ofaf048. doi: 10.1093/ofid/ofaf048 (PMC11800476; doi:10.1093/ofid/ofaf048)
Supplement: ofaf048_Supplementary_Data [file ofaf048_supplementary_data.zip › SymptomsAndFunctionalLimitationsQuestionnaire.pdf]

Symptoms And Functional Limitations

Record ID ______________________________

Use this worksheet to document the participant's symptoms and functional limitations for each day of the assessment.

Time Point:
- ◯ 7-14 days
- ◯ 1 month
- ◯ 3 months
- ◯ 6 months
- ◯ 12 months
- ◯ 2 years
- ◯ 3 years
- ◯ floating visit

Participant ID code: ______________________________

Interviewer's initials: ______________________________

Date form completed ______________________________

Date of COVID diagnosis/qualifying COVID test: [pcr_date]

A. Instructions to Staff

Read this form prior to administering the questions to participants. You should read the text, including questions and response options, exactly as written. You may provide clarifying questions if asked by participants, but attempt to use the language provided on this form whenever possible.

For the symptom assessment, the goal is to assess the experience of the symptom when measured from the participant's perspective (so it is a "patient-reported outcome"). Each question related to a symptom should reflect the worst status of the participant's currnet experience.

For the assessment of usual social or functional activity, there are two main questions. First, the type of activity that is limited due to COVID-19. Second, the degree of interference or limitation in the activity. When more than one activity is interfered with, the degree of interference should reflect the worst or most severe limitation.

B. Symptom Assessment

Read the following to the participant:

The following questions will assess symptoms that you may still be having since becoming ill from COVID-19. These include any symptoms that were not present prior to becoming ill, as well as symptoms that may have been present prior to your illness but are now worse. If a symptom was present prior to getting COVID and is unchanged, that would not be a new symptom that we will collect. Any questions?

-> Instructions to staff: Address any questions prior to proceeding.

---

1. Among the following options, which best represents the degree to which you have recovered or returned to your usual state of health; that is, your health prior to having COVID:

◯ Completely recovered; select this only if you have no lingering symptoms at all from COVID and can conduct usual activities without limitations
◯ Mostly recovered
◯ Somewhat recovered
◯ Not at all recovered

---

1. a. What month and year did your last symptoms resolve?	____________________ (example: JUN 2021)
Select the first date of the month/year on the calendar when symptoms were resolved.	____________________

---

1. b. Which of the following describes the type of symptoms that lasted the longest during your recovery from COVID?

◯ fatigue or energy level
◯ repiratory symptoms
◯ gastrointestinal symptoms
◯ trouble thinking, concentrating, or "brain fog"
◯ mental health symptoms
◯ other

Other:	____________________
2. In the past week, have you had any of the following symptoms due to COVID-19?<br><br>a. Loss of taste	◯ No ◯ Yes
b. Loss of smell	◯ No ◯ Yes
c. Nasal congestion	◯ No ◯ Yes
d. Runny nose	◯ No ◯ Yes
e. Sore throat	◯ No ◯ Yes
f. Hair loss	◯ No ◯ Yes

3. I'm now going to review a list of symptoms. Remember, this is only for symptoms that are continuing since you became ill with COVID-19. We want you to indicate the worse severity at any point during the previous week. For each symptom I want you to state if you experienced the symptom, and if so, then rate the severity as "mild," "moderate," or "severe." The severity is from your experience and represents how bothersome the symptom is.

a. Feeling feverish

◯ Not experiencing ◯ Mild ◯ Moderate ◯ Severe

b. Fatigue

◯ Not experiencing ◯ Mild ◯ Moderate ◯ Severe

c. Headache

◯ Not experiencing ◯ Mild ◯ Moderate ◯ Severe

d. Cough

◯ Not experiencing ◯ Mild ◯ Moderate ◯ Severe

e. Difficulty breathing

◯ Not experiencing ◯ Mild ◯ Moderate ◯ Severe

f. Chest pain

◯ Not experiencing ◯ Mild ◯ Moderate ◯ Severe

g. Body aches

◯ Not experiencing ◯ Mild ◯ Moderate ◯ Severe

h. Abdominal pain

◯ Not experiencing ◯ Mild ◯ Moderate ◯ Severe

i. Nausea with or without vomiting

◯ Not experiencing ◯ Mild ◯ Moderate ◯ Severe

j. Diarrhea

◯ Not experiencing ◯ Mild ◯ Moderate ◯ Severe

k. Skin rash, even if intermittent

◯ Not experiencing ◯ Mild ◯ Moderate ◯ Severe

l. Hair loss

◯ Not experiencing ◯ Mild ◯ Moderate ◯ Severe

m. Difficulty concentrating or inability to focus

◯ Not experiencing ◯ Mild ◯ Moderate ◯ Severe

n. Confusion or difficulty thinking

◯ Not experiencing ◯ Mild ◯ Moderate ◯ Severe

o. Memory loss or forgetfulness

◯ Not experiencing ◯ Mild ◯ Moderate ◯ Severe

p. Difficulty sleeping

◯ Not experiencing ◯ Mild ◯ Moderate ◯ Severe

q. Excessive or daytime sleepiness

◯ Not experiencing ◯ Mild ◯ Moderate ◯ Severe

r. Depressed mood

◯ Not experiencing ◯ Mild ◯ Moderate ◯ Severe

s. Anxiety, including anxious feelings that come and go

◯ Not experiencing ◯ Mild ◯ Moderate ◯ Severe

t. Other

◯ Not experiencing ◯ Mild ◯ Moderate ◯ Severe

Specify:

____

C. Interference with Social or Functional Activity

Read the following to the participant:
The following questions are intended to assess whether COVID is interfering with activities in your life.
We would like you to consider three things when determining this:
• First, the activity limitation should be related to a symptom that is continuing since COVID
• Second, the activity limitation should also be new or worse since COVID. For example, if you were unable to do something prior to becoming ill, the activity would now need to be more difficult.
• Third, a limitation just means you cannot do the activity like you previously were able to. This could mean that you can no longer do the activity or just that it is more difficult. For example, you may only be able to do the activity for a shorter duration, or less often, or something for which you now need help.

1. For each option below, indicate whether COVID symptoms are interfering with or limiting your ability to perform one or more of the activities below. (Mark all that apply.)

- ☐ Social interactions; such as talking or spending time with family or friends, including by video or phone.
- ☐ Physical activity; this may include something simple like walking around the house or something more significant like exercise
- ☐ Work or job function; such as reduced hours or reduced ability to perform job tasks due to your health
- ☐ Usual activities of daily living; these are activities such as dressing, eating or cooking, walking independently, using a bathroom, showering/bathing and other examples of personal hygiene
- ☐ None of the previous examples

2.a. If you consider the social interactions that are limited by your current COVID symptoms, indicate which of the following best reflects the degree of the interference when compared to your pre-illness state.

- ◯ a. Activity occurs less often than usual
- ◯ b. Activity is more difficult but still able to perform
- ◯ c. Activity requires significant modification to perform
- ◯ d. Activity requires significant assistance to perform
- ◯ e. Activity can no longer be performed

2.b. If you consider the physical activity that is limited by your current COVID symptoms, indicate which of the following best reflects the degree of the interference when compared to your pre-illness state.

- ◯ a. Activity occurs less often than usual
- ◯ b. Activity is more difficult but still able to perform
- ◯ c. Activity requires significant modification to perform
- ◯ d. Activity requires significant assistance to perform
- ◯ e. Activity can no longer be performed

2.c. If you consider the work or job function that is limited by your current COVID symptoms, indicate which of the following best reflects the degree of the interference when compared to your pre-illness state.

- ◯ a. Activity occurs less often than usual
- ◯ b. Activity is more difficult but still able to perform
- ◯ c. Activity requires significant modification to perform
- ◯ d. Activity requires significant assistance to perform
- ◯ e. Activity can no longer be performed

2.d. If you consider the usual activities of daily living that is limited by your current COVID symptoms, indicate which of the following best reflects the degree of the interference when compared to your pre-illness state.

- ◯ a. Activity occurs less often than usual
- ◯ b. Activity is more difficult but still able to perform
- ◯ c. Activity requires significant modification to perform
- ◯ d. Activity requires significant assistance to perform
- ◯ e. Activity can no longer be performed
